# Postprandial hyperglycemia and endothelial function in type 2 diabetes: focus on mitiglinide

**DOI:** 10.1186/1475-2840-11-79

**Published:** 2012-06-29

**Authors:** Lisa Kitasato, Taiki Tojo, Yuko Hatakeyama, Ryo Kameda, Takehiro Hashikata, Minako Yamaoka-Tojo

**Affiliations:** 1 Kitasato University Graduate School of Medical Sciences, Sagamihara, Japan; 2 Department of Cardioangiology, Kitasato University School of Medicine, Sagamihara, Japan; 3 Special Department of Cardiac Rehabilitation and Preventive Cardiovascular Medicine, Kitasato University School of Medicine, Sagamihara, Japan; 4 Kitasato University School of Allied Health Sciences, Sagamihara, Japan; 5 Department of Rehabilitation, Kitasato University School of Allied Health Sciences, 1-15-1 Kitasato, Minami-ku, Sagamihara, Kanagawa, 252-0373, Japan

**Keywords:** Atherosclerosis, Endothelial function, Cardiovascular disease prevention

## Abstract

The risk of cardiovascular complication in a diabetes patient is similar to that in a nondiabetic patient with a history of myocardial infarction. Although intensive control of glycemia achieved by conventional antidiabetic agents decreases microvascular complications such as retinopathy and nephropathy, no marked effect has been reported on macrovascular complications or all-cause mortality. Evidence from VADT, ACCORD, and ADVANCE would suggest that glycemic control has little effect on macrovascular outcomes. Moreover, in the case of ACCORD, intensive glycemic control may be associated with an increased risk of mortality. There is sufficient evidence that suggests that postprandial hyperglycemia may be an independent risk factor for cardiovascular disease in diabetes patients. However, there are no prospective clinical trials supporting the recommendation that lowering postprandial blood glucose leads to lower risk of cardiovascular outcomes. Mitiglinide is a short-acting insulinotropic agent used in type 2 diabetes treatment. It has a rapid stimulatory effect on insulin secretion and reduces postprandial plasma glucose level in patients with type 2 diabetes. Because of its short action time, it is unlikely to exert adverse effects related to hypoglycemia early in the morning and between meals. Mitiglinide reduces excess oxidative stress and inflammation, plays a cardioprotective role, and improves postprandial metabolic disorders. Moreover, mitiglinide add-on therapy with pioglitazone favorably affects the vascular endothelial function in type 2 diabetes patients. These data suggest that mitiglinide plays a potentially beneficial role in the improvement of postprandial hyperglycemia in type 2 diabetes patients and can be used to prevent cardiovascular diseases. Although the results of long-term, randomized, placebo-controlled trials for determining the cardiovascular effects of mitiglinide on clinical outcomes are awaited, this review is aimed at summarizing substantial insights into this topic.

## Vascular endothelial function as a surrogate marker for atherosclerosis progression

Endothelial dysfunction occurs in diabetes patients, especially in chronic hyperglycemic condition [1-3]. Noninvasive testing of endothelial function for cardiovascular risk stratification has been the “holy grail” of Cardiology for some time [4]. Unfortunately, because of technical difficulties, the measurement of shear stress-induced flow-mediated dilatation (FMD) of the brachial artery by arterial ultrasound was used to evaluate vascular endothelial function and is not yet commonly practiced in large-scale clinical trials [5,6]. Instead of FMD, new techniques have recently been proposed as potentially applicable screening tools for evaluating vascular endothelial function in humans. A novel noninvasive vascular test by pulse amplitude tonometry (PAT) before and after reactive hyperemia (RH) is particularly noteworthy [7]. Measuring digital RH-PAT involves quantifying arterial pulsatile volume at rest and during a condition of increased shear stress that results in the release of nitric oxide (NO) [8]. At least 50% of RH is dependent on endothelial NO production [9]. PAT is performed before and after a 5-min ischemic stress, generating an RH-PAT index, which is normalized with respect to the control arm [10]. According to a previous clinical study, 94 patients without obstructive coronary artery disease and with/without coronary microvascular endothelial dysfunction were examined using RH-PAT; the average RH-PAT index was lower in patients with coronary endothelial dysfunction than in those with normal coronary endothelial function (1.27 ± 0.05 vs 1.78 ± 0.08; *P* < 0.001) [7].

Clinical studies using the PAT probe have suggested altered endothelial function in children with cardiovascular risk of type 1 diabetes [11]. Further, impaired RH-PAT responses have been shown to be inversely related to multiple risk factors, particularly diabetes, obesity, high cholesterol concentrations, and smoking, in a group of nearly 2000 subjects in the Framingham Third Generation Cohort study [8]. In a recent randomized controlled trial (RCT) using the RH-PAT technique, 35 patients with obesity hypoventilation syndrome were randomized to either the noninvasive ventilation (NIV) group or the control group represented by lifestyle counseling [12]. After 1 month of NIV treatment, sleep and blood gas measurements had improved markedly but the inflammatory, metabolic, and cardiovascular markers were not affected. Accordingly, neither RH-PAT nor arterial stiffness improved [12]. Probably, the intervention and/or the study period may be insufficient to improve endothelial function and other biomarkers. In our previous study, 4-week intervention for increasing physical activity had significantly improved endothelial function in high-risk patients with cardiovascular disease [13].

## Why does atherosclerosis progress in type 2 diabetes patients?

Type 2 diabetes (non-insulin-dependent diabetes) is an important risk factor in atherosclerotic cardiovascular disease [14,15]. It is a chronic disease, affecting 346 million people worldwide [16]. In many cases, type 2 diabetes is preceded by a symptom-free period of impaired glucose tolerance (IGT) and/or impaired fasting glucose (IFG), which are characterized by chronic hyperglycemia with prediabetic condition. Hyperglycemia plays a specific role in atherosclerosis progression in patients with diabetes and IGT [17]. Needless to say, control of other cardiovascular risk factors, such as blood pressure, dyslipidemia, and smoking, have a more significant impact than glycemic control in patients with type 2 diabetes [18].

Endothelial dysfunction is the initial step in atherosclerosis and occurs in patients with chronic hyperglycemia [17,19-23]. In hyperglycemia patients, oral glucose loading rapidly suppresses endothelial-dependent vasodilatation through increase in the production of oxygen-derived free radicals [24]. In patients with diabetes and IGT, hyperglycemia is usually associated with other coronary risk factors, such as dyslipidemia, hypertension, and obesity. These factors are also known to cause endothelial dysfunction [25-27]. Atherosclerosis-induced coronary artery disease is the main cause of morbidity and mortality in diabetic patients [28].

Endothelial dysfunction is a key component of atherosclerosis and contributes to the development of clinical cardiovascular disease [29]. In the presence of vascular risk factors, vascular endothelial cells undergo phenotypic changes resulting in decreased nitric oxide bioactivity, thereby promoting vasoconstriction, vascular inflammation, endothelial-mesenchymal transition, and thrombosis [30-32]. Coronary risk factors are associated with impaired vasomotor function, and individuals with abnormal vasodilator function have increased cardiovascular event rates [33]. Obesity and diabetes, along with the associated dyslipidemia and insulin resistance, have been linked to impaired vasodilator responses in humans [34-37] and animal models [38-40].

## What are the possible effects of antidiabetic agents?

Fatal and nonfatal macrovascular events induced by type 2 diabetes are the main reasons for decreased life expectancy; it is about 8 years shorter in a 40-year-old patient newly diagnosed with diabetes than in the general population [41]. The risk of a cardiovascular complication in a diabetes patient is similar to that in a nondiabetic patient with a history of myocardial infarction [42]. Intensive control of glycemia with conventional antidiabetic agents decreases microvascular complications such as retinopathy and nephropathy, but it has no marked effect on macrovascular complications or all-cause mortality [43,44].

Substantial evidence suggests that postprandial hyperglycemia may be an independent risk factor for cardiovascular disease in diabetes patients [42,45-48]. Hyperglycemia acutely increases the levels of circulating proinflammatory cytokines by an oxidative mechanism, and the effects are pronounced in subjects with IGT and diabetes [49]. Excessive oxidative stress caused by imbalance between free-radical production and antioxidant response, followed by systemic vascular inflammation, is involved in the pathogenesis of cardiovascular disease in diabetes patients [50-52]. Considerable epidemiological and clinical studies, such as the Funagata study and the Diabetes Epidemiology Collaborative analysis of Diagnostic criteria in Europe (DECODE) study, have established that even a prediabetic state, including IGT, is strongly associated with the occurrence of cardiovascular diseases [45,53,54]. Postprandial acceleration of oxidative stress and inflammation has been observed in patients with type 2 diabetes [49,55-58]. The reduction in carotid intima-media thickness was associated with the improvement of postprandial but not fasting hyperglycemia [59]. Therefore, treating postprandial hyperglycemia may have a positive effect on atherosclerosis progression and cardiovascular diseases.

We believe that antidiabetic agents can prevent cardiovascular events by improving hyperglycemia in both preprandial and postprandial conditions, which may inhibit atherosclerosis progression caused by hyperglycemia-induced oxygen-derived free radicals [60]. Postprandial hyperglycemia is an important target to prevent cardiovascular events [61]. Moreover, postprandial dysmetabolism (hyperglycemia and hyperlipidemia) is associated with increased inflammation, endothelial dysfunction, decreased fibrinolysis, plaque instability, and cardiac events, even in nondiabetic patients [62].

By targeting mainly postprandial hyperglycemia, glinide drugs and alpha-glucosidase inhibitors (α-GI) favorably affect several cardiovascular risk factors such as obesity, dyslipidemia, hypertension, and high glycemic variability with little or no risk of hypoglycemia. Improving postprandial hyperglycemia by acarbose favorably affects endothelial function and carotid intima-media thickening in humans and improves cardiac interstitial fibrosis and hypertrophy of cardiomyocytes in animal models [63].

When antidiabetic monotherapy is not sufficient to achieve the desired therapeutic effect, other drugs, such as metformin, sulfonylureas, glinides, α-GI, thiazolidinediones, glucagon-like peptide-1 receptor agonists, dipeptidyl peptidase-4 inhibitors, and insulin, can be added to the treatment regimen [64,65].

Pioglitazone is an antidiabetic agent and agonist of peroxisome proliferator-activated receptor-γ (PPAR-γ); it improves insulin resistance and is widely used in type 2 diabetes treatment to prevent cardiovascular disorders [66,67]. Compared to placebo, pioglitazone reduced the risk of conversion of impaired glucose tolerance to type 2 diabetes by 72%, but it was associated with significant weight gain and edema [68]. Bladder cancer episodes significantly increased after pioglitazone administration in rodents and humans [69], which may trigger the growth of bladder tumors by increasing the local expression of vascular endothelial growth factors [70]. Therefore, physicians tend to prefer a combination therapy with pioglitazone and other anti-diabetic agent to high-dose pioglitazone monotherapy. One of the best options may be mitiglinide add-on therapy with the standard dose of pioglitazone, in which mitiglinide overcomes the insufficiency of pioglitazone to improve postprandial hyperglycemia in type 2 diabetes patients.

Although the results of long-term, randomized, placebo-controlled trials for determining cardiovascular effects of antidiabetic agents in terms of clinical outcomes are awaited, herein, we aimed to summarize substantial insights about mitiglinide.

## Mitiglinide, an immediate short-acting insulinotropic agent

Asian population is more insulin resistance than others [71]. Asian patients with type 2 diabetes showed several characteristic features such as high insulin resistance with low BMI and relatively young age at diagnosis [72]. Mitiglinide, an insulinotropic sulfonylurea (SU) receptor ligand, is a benzylsuccinic acid derivative developed in Japan. It is an insulin secretagogue that acts on pancreatic β-cells, and unlike other SU agents, it has rapid action and short action time [73-77]. Its preprandial administration controls postprandial hyperglycemia and improves overall glycemic control [78]. Because of its short action time, it is unlikely to exert hypoglycemic adverse effects early in the morning and between meals. Therefore, mitiglinide may become a first choice drug for the early stage of type 2 diabetes [79].

Mitiglinide exerts selective action on the ATP-dependent K channel (K_ATP_) channel (Kir6.2/SUR1) of pancreatic β-cells and has stronger affinity to the channel than other insulinotropic SU receptor ligands, namely, repaglinide and nateglinide [80]. In addition to its glucose-lowering effect, mitiglinide inhibits postprandial hypertriglyceridemia in OLETF (Otsuka Long-Evans Tokushima Fatty) rats, which exhibit insulin resistance and visceral fat accumulation and are considered as aging diabetes models [81]. Mitiglinide also improves postprandial hyperglycemia in type 2 diabetes patients via both an insulin-mediated indirect effect on the liver and a direct regulatory influence over hepatic glucose metabolism [82].

## Mitiglinide ameliorates postprandial hyperglycemia

Compared to persistent hyperglycemia, intermittent hyperglycemia, ie, glucose spikes, induces apoptosis of vascular endothelial cells [83]. Mitiglinide shows rapid stimulatory effect on insulin secretion and reduces postprandial plasma glucose levels in type 2 diabetes patients [73-75]. In type 2 diabetes patients, mitiglinide improved postprandial glucose levels, but it had no effect on increased serum adiponectin levels or decreased urinary albumin excretion [84].

Mitiglinide reduces the levels of circulating biomarkers of oxidative stress and inflammation caused by postprandial hyperglycemia [85]. In a meal test in 40 diabetic patients, 10 mg mitiglinide administration stimulated rapid insulin secretion, accompanied by reduction of postprandial hyperglycemia [85]. The study showed that controlling postprandial hyperglycemia with mitiglinide significantly improved the levels of oxidative stress and inflammation markers that are increased in the postprandial state in diabetic patients [85].

Mitiglinide administration also decreased free fatty acids (FFA) levels at 60 min after a meal tolerance test in type 2 diabetes patients [86]. Mitiglinide significantly lowered hemoglobin A1c (HbA1c) levels and increased 1,5-anhydroglucitol levels after 6 months and significantly decreased urinary albumin levels after 12 months [86]. These data suggest that mitiglinide certainly improved postprandial hyperglycemia, which is crucial to treating metabolic disorders, including insulin resistance and dyslipidemia, in type 2 diabetes patients.

## Mitiglinide has cardioprotective effects

The term *meglitinide analogs* was introduced in 1995 to cover new molecules proposed as non-sulfonylurea insulinotropic agents that had structural analogy with meglitinide, such as repaglinide, nateglinide, and mitiglinide [87]. Results of the STOP-NIDDM trial [61] suggest that meglinide analogs (glinide drugs) may help protect type 2 diabetes patients against cardiovascular events [88]. If mitiglinide could even partly regulate oxidative stress and vascular inflammation, it could be used to prevent cardiovascular diseases. Mitiglinide treatment significantly reduced plasma nitrityrosine, malondialdehyde (MDA), and oxidant LDL (oxLDL) levels [85]. Furthermore, mitiglinide administration preserved plasma total radical-trapping antioxidant parameter (TRAP) compared with placebo. Importantly, mitiglinide decreased the levels of proinflammatory cytokines such as interleukin (IL)-6, IL-18, and tumor necrosis factor (TNF)-α [85]. Although the study only examined the effect of acute administration of mitiglinide, it appears that reducing postprandial oxidative stress and inflammation may result in long-term effects of cardiovascular prevention in diabetic patients.

Postprandial insulin secretion is considered to increase cystatin C levels, which provide an accurate estimate of renal function in diabetic patients. In 19 Japanese diabetes patients, 3-month mitiglinide monotherapy increased cystatin C levels and had no effect on hs-CRP levels [89]. Hence, there is very less evidence to judge the controversy.

Although the use of SU agents in type 2 diabetes patients has been quite common, increased risk of cardiovascular complications and increased fatality rate after myocardial infarction have been reported in these patients [90]. Sulfonylurea receptors (SUR), which constitute the K_ATP_ channel, have 3 subtypes with differing distributions: SUR1 (present in pancreatic β cells), SUR2A (cardiac myocytes), and SUR2B (vascular smooth muscle cells) [91,92]. Hence, *in vitro* selectivity of the insulin secretagogues may be important for the cardiovascular outcome of diabetic patients with coronary artery disease [91]. In isolated perfused rat hearts, mitiglinide (selective blocker for SUR1) preserved the cardioprotective effect of ischemic preconditioning compared to glibenclamide (nonselective SUR blocker) [93]. In another study on isolated perfused rat hearts, glibenclamide induced a significant increase in left ventricular end-diastolic pressure and significantly decreased the left ventricular systolic/developed pressure, and glimepiride induced significant decrease in the left ventricular developed pressure [94]. However, mitiglinide had no effects on canine isolated coronary arteries or perfused rat hearts. Thus, mitiglinide may be safer than glibenclamide and glimepiride with regard to its cardiovascular effects in diabetes patients.

## Effect of mitiglinide on endothelial function

Evidence from previous studies suggests the importance of the association between postprandial hyperglycemia and endothelial dysfunction in diabetes patients. The outcomes of 12-week interventions of 300 mg/day acarbose, 270 mg/day nateglinide, or no medication were compared among 3 groups (10 subjects in each group) of patients with new-onset type 2 diabetes [95]. Only acarbose improved postprandial endothelial function, as assessed by %FMD at 0 and 120 min after a cookie test.

**Figure 1:**
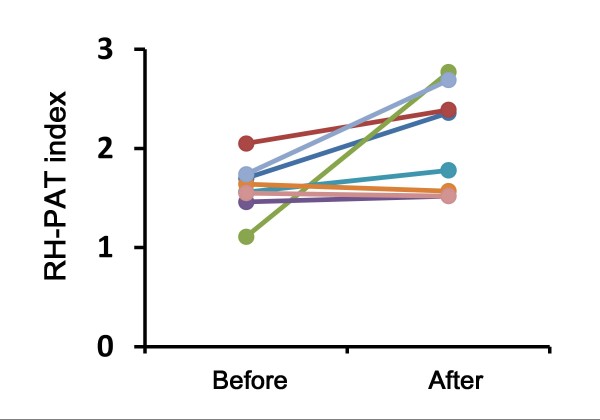
**Effect of mitiglinide add-on therapy on endothelial function.** Reactive hyperemia-peripheral arterial tonometry (RH-PAT) index was measured for evaluating vascular endothelial function in type 2 diabetes patients before and after the 12-week mitiglinide add-on therapy.

The effect of mitiglinide with pioglitazone on endothelial function remains largely unexplored. To investigate whether mitiglinide could improve endothelial function, we performed a pilot study on mitiglinide add-on therapy with pioglitazone on type 2 diabetes patients who were insufficiently controlled with pioglitazone monotherapy. In 8 patients with type 2 diabetes mitiglinide was administered as add-on therapy with 10 mg/day pioglitazone for 12 weeks. Endothelial function was measured with the RH-PAT (Itamar) technique in all patients before and after the intervention [96,97]. Mitiglinide add-on therapy significantly improved the RH-PAT index from 1.6 ± 0.3 to 2.1 ± 0.5 (*P* = 0.0373) in diabetes patients with pioglitazone monotherapy (Figure 1). Significant reduction of HbA1c (Japan Diabetes Society [JDS]) levels from 6.4% ± 0.3% to 6.1% ± 0.4% (*P* = 0.0062) was also observed without any other modification of lifestyle or other drug administrations; however, there was no reduction in triglyceride level, LDL cholesterol level, body weight, or waist circumference. In type 2 diabetes patients, combination therapy with mitiglinide and pioglitazone resulted in marked improvements in HbA1c levels and endothelial function. In 16 patients with type 2 diabetes treated with 30 mg/day mitiglinide, levels of plasma glucose, FFA, and urinary albumin excretion were significantly decreased [86]. One of the mechanisms may be the reduction of circulating levels of FFA by mitiglinide administration.

## Effect of mitiglinide in randomized clinical trials

Although there has been no large-scale RCT on mitiglinide monotherapy or mitiglinide add-on therapy with other antidiabetic drugs thus far, several RCTs for mitiglinide therapy have been reported. In this review, we have summarized the findings of these papers, which suggest that the use of mitiglinide for type 2 diabetes treatment can be assessed in future RCTs.

Several RCTs for mitiglinide monotherapy have been reported. A multicenter, double-blind, randomized study comparing mitiglinide with nateglinide in 291 Chinese type 2 diabetes patients reported that 10 to 20 mg mitiglinide 3 times daily exerted blood glucose control similar to 120 mg nateglinide 3 times daily [98].

Recently, an RCT of nateglinide, but not mitiglinide, was reported from Japan. The diabetes and diffuse coronary narrowing (DIANA) study is a prospective, randomized, open-label multicenter trial [99]. Japanese patients (n = 302) with coronary artery disease and IGT/diabetes were randomly assigned to lifestyle intervention only, voglibose, or nateglinide groups and their 1-year coronary atherosclerotic changes were evaluated using quantitative coronary arteriography. Although coronary atherosclerotic changes were similar for voglibose and nateglinide, an improvement in glycemic status after 1 year was associated with decreased atheroma progression, regardless of the treatment [99]. These data suggest that improving postprandial hyperglycemia by lifestyle modification, α-GI, or glinide drugs can prevent coronary artery progression.

There are 2 RCTs of mitiglinide add-on treatment with pioglitazone. In a 16-week, multicenter, randomized, double blind, parallel-group study, mitiglinide administration with pioglitazone monotherapy improved glycemic control in 381 Japanese patients with type 2 diabetes [100]. HbA1c level at final evaluation was 7.43% ± 0.97% in the pioglitazone monotherapy group and 6.84% ± 0.7in the mitiglinide 10 mg group [100]. In type 2 diabetic patients, the combination therapy for 1 year improved HbA1c, fasting plasma glucose, and postprandial plasma glucose levels [101].

## How can mitiglinide be used to prevent cardiovascular disease?

The management of high-risk patients with diabetes includes not only diet and exercise but also a combination of antihyperglycemic treatment with lipid-lowering, antihypertensive, and antiplatelet therapy [60]. Numerous agents with different mechanisms of action and different pharmacological profiles are being used with the aim of improving glycemic control in patients with diabetes. How do we select an appropriate combination of drugs for antidiabetic therapy?

In case of advanced diabetes, combination therapy with mitiglinide and insulin glargine, which has a 24-h time-action profile with no pronounced peak [102,103], may be a useful regimen to lower postprandial hyperglycemia before switching to high-dose of SUs or intensive insulin therapy using insulin injections [104].

Furthermore, 10 mg of mitiglinide once a day at lunchtime to twice daily injections of premixed insulin are effective for type 2 diabetes treatment.

Especially for obese patients, the combination of calorie restriction and mitiglinide administration lowered the visceral fat area and body weight, whereas glimepiride with restricted diet did not significantly reduce visceral fat area or body weight [105]. These findings suggest that combined use of mitiglinide with calorie restriction is warranted in patients with obesity and/or metabolic syndrome, and that short-acting oral hyperglycemic agents and exogenous short-acting insulin are useful for those attempting to undergo lifestyle modification [105].

The therapeutic options for patients with type 2 diabetes and chronic kidney disease (CKD) are limited because decreased glomerular filtration rate results in the accumulation of certain drugs and/or their metabolites [106]. With careful monitoring of hypoglycemia, mitiglinide can be safely used for diabetic CKD patients on hemodialysis (HD) [107]. Moreover, combination therapy of mitiglinide and voglibose may have the potential for treating diabetes patients on HD [108]. In these cases, mitiglinide administration should be initiated at a lower dose by monitoring glycemic control. In non-HD CKD patients, repeated asking about hypoglycemic symptoms and adverse events may be most important and useful to monitor the presence of hypoglycaemia [109].

**Figure 2:**
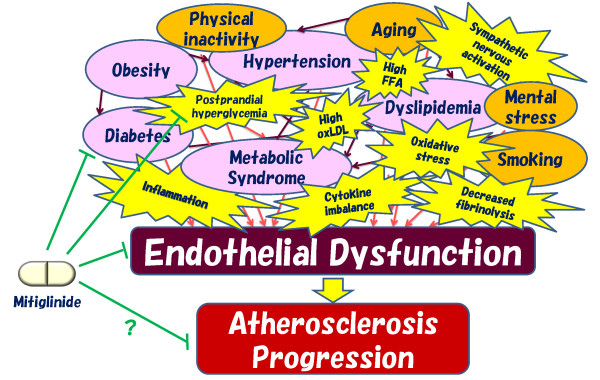
**The effects of mitiglinide on endothelial function and preventing cardiovascular disease.** Multiple risk factors induce vascular endothelial dysfunction and the subsequent atherosclerosis progression. FFA, free fatty acids; oxLDL, oxidized low-density lipoprotein.

The above findings indicate that in type 2 diabetes patients at high risk of coronary artery disease, combination therapy with agents that improve vascular endothelial function, such as mitiglinide and pioglitazone, is a promising therapeutic strategy for total risk management of cardiovascular disease prevention (Figure 2).

## Conclusions

In conclusion, we reviewed the effects of mitiglinide on postprandial hyperglycemia and vascular endothelial function in type 2 diabetes patients. The results of long-term RCTs for the ultimate determination of the cardiovascular effects of mitiglinide in terms of clinical outcomes are awaited; however, the improvement of postprandial hyperglycemia may be crucial to prevent atherosclerosis progression and cardiovascular events.

## Abbreviations

ACCORD trial: The action to control cardiovascular risk in diabetes trial; ADVANCE trial: The action in diabetes and vascular disease: preterax and diamicron modified release controlled evaluation trial; α-GI: Alpha-glucosidase inhibitors; ATP: Adenosine triphosphate; BMI: Body mass index; CKD: Chronic kidney disease; DECODE: Diabetes epidemiology collaborative analysis of diagnostic criteria in Europe; DIANA study: The diabetes and diffuse coronary narrowing study; FFA: Free fatty acids; FMD: Flow-mediated dilatation; HbA1c: Hemoglobin A1c; HD: Hemodialysis; hs-CRP: High sensitivity C-reactive protein; IFG: Impaired fasting glucose; IGT: Impaired glucose tolerance; IL: Intereukin; K_ATP_: The ATP-dependent potassium channel; Kir: Inwardly rectifying potassium channel; LDL: Low-density lipoprotein; MDA: Malondialdehyde; NIV: Noninvasive ventilation; NO: Nitric oxide; OLETF rats: Otsuka long-evans tokushima fatty rats; oxLDL: Oxidized LDL; PAT: Peripheral arterial tonometry; PPAR: Peroxisome proliferator-activated receptors; RCT: Randomized controlled trial; RH-PAT: Reactive hyperemia-PAT; STOP-NIDDM trial: Study to prevent non-insulin-dependent diabetes mellitus trial; SU: Sulfonylurea; SUR: Sulfonylurea receptor; TNF: Tumor necrosis factor; TRAP: Total-trapping antioxidant parameter; VADT: The veterans affairs diabetes trial.

## Competing interests

The authors declare that they have no competing interests.

## Authors' contributions

LK participated in the preparation of the manuscript. TT co-designed the review. YH collected data. MYT wrote the final manuscript. All authors have read and approved the final manuscript.
